# Preclinical Evidence and Possible Mechanisms of Extracts or Compounds from Cistanches for Alzheimer’s Disease

**DOI:** 10.14336/AD.2018.0815-1

**Published:** 2019-10-01

**Authors:** Xiao-Li Zhou, Meng-Bei Xu, Ting-Yu Jin, Pei-Qing Rong, Guo-Qing Zheng, Yan Lin

**Affiliations:** Department of Neurology, the Second Affiliated Hospital and Yuying Children's Hospital of Wenzhou Medical University, Wenzhou, China

**Keywords:** Cistanches, Alzheimer’s disease, dementia

## Abstract

Currently, disease-modified strategies to prevent, halt or reverse the progress of Alzheimer’s disease (AD) are still lacking. Previous studies indicated extracts or compounds from Cistanches (ECC) exert a potential neuroprotective effect against AD. Thus, we conducted a preclinical systematic review to assess preclinical evidence and possible mechanisms of ECC in experimental AD. A systematical searching strategy was carried out across seven databases from their inceptions to July 2018. Twenty studies with 1696 rats or mice were involved. Neurobehavioral function indices as primary outcome measures were established by the Morris water maze test (n = 11), step-down test (n = 10), electrical Y-maze test (n = 4), step-through test (n = 3), open field test (n = 2) and passage water maze test (n = 1). Compared with controls, the results of the meta-analysis showed ECC exerted a significant effect in decreasing the escape latency, error times and wrong reaction latency in both the training test and the retention test, and in increasing the exact time and the percentage of time in the platform-quadrant and the number of platform crossings (all *P*<0.01). In conclusion, ECC exert potential neuroprotective effects in experimental AD, mainly through mechanisms involving antioxidant stress and antiapoptosic effects, inhibiting Aβ deposition and tau protein hyperphosphorylation and promoting synapse protection. Thus, ECC could be a candidate for AD treatment and further clinical trials.

Alzheimer’s disease (AD) is one of the most common, stubborn neurodegenerative disorders and is characterized by progressive cognitive dysfunction and behavioral impairment [1-3], accounting for 60~80% of all dementia cases [4]. In 2017, 5.3 million Americans aged 65 and older lived with AD; by 2050, this number will rise as high as 16 million in the US and 135 million worldwide. More than 15 million Americans provided an estimated 18.2 billion hours of unpaid care for patients with dementia, valued at more than $230 billion; by 2050, these costs could rise as high as $1.1 trillion [5, 6]. However, the cause of AD remains poorly understood. It is widely accepted that AD is associated with extracellular deposits of amyloid β (Aβ) peptide and intracellular tau aggregates [7, 8]. As apoptosis of neurons develops and connections among cells are lost, learning and memory impairment emerges and disease progresses [9, 10]. The prescription drugs approved by the FDA in the US for AD symptom control include: (1) cholinesterase inhibitors (ChEIs) such as donepezil, galantamine, rivastigmine and huperzine A, which maintain average acetylcholine levels by reducing the activity of acetylcholinesterase and (2) N-methyl-D-aspartic acid (NMDA) receptor antagonist, memantine, which protects neurons against excessive glutamate by partially blocking NMDA receptors [11]. However, all of them are only temporarily symptom relievers and can bring undesirable side effects, such as headache, dizziness, nausea, vomiting, insomnia, other somatic symptoms and drug interactions [11-13]. Disease-modified strategies to prevent, halt or reverse AD progress are urgently needed. There are rising numbers of AD patients seeking various kinds of complementary and alternative medicines worldwide, among which Chinese herbal medicines (CHMs) have high potential [14].

*Herba Cistanches*, a desert living Cistanche, *Roucongrong*, the dried fleshy stem of *Cistanche deserticola Y. C. Ma*, first recorded in *Shennongbencaojing* (*Shennong’s Classic of Materia Medica,* written about 475 B.C.-220 A.D.), is known as the desert ginseng and is of high medicinal value [15, 16]. Showing a high antioxidative and antiinflammatory activity, Cistanches possess broad medicinal functions in neuroprotection, immunomodulation, endocrine regulation, hepatoprotection and bone-formation promotion. Nowadays, Cistanches is widely used in CHM formulas for treating various kinds of disorders, including aging and dementia [17-20]. Extracts or compounds from Cistanches (ECC), containing or representing the major bioactive ingredients, include Cistanches deserticola polysaccharides (CDPS), glycosides of Cistanches (GCs), and phenylethanoid glycosides (PhGs) such as echinacoside (ECH), acteoside (AS) and tubuloside B [21, 22]. Some preliminary clinical trials [23-26] indicated that ECC monotherapy for AD symptom control encouragingly received positive feedbacks. However, the effects of ECC and possible mechanisms behind these effects on AD remain uncertain. Furthermore, the clinical study is limited owing to various restrictions due to morality and methodology [27]. The systematic evaluation of preclinical researches is an essential method to integrate preclinical evidence and can be of high value in improving the quality of preclinical researches and guiding potential clinical translation and application [28, 29]. Thus, in the present study, we aim to conduct a preclinical systematic review of the efficacy of ECC and the mechanisms involved in experimental AD.

## MATERIALS AND METHODS

### Search strategies

Seven English and Chinese databases, including PubMed, the Cochrane Library, EMBASE, China National Knowledge Infrastructure (CNKI), VIP Journals Database, China Biology Medicine Database (CBM) and Wanfang Database, were electronically searched from their inceptions to July 2018. The following keywords were used: “Cistanche* OR *Roucongrong*” and “Alzheimer’s disease OR dementia OR mild cognitive impairment”. All studies were limited to animals.

### Eligibility criteria

#### Types of studies

Animal studies that assess the effectiveness of ECC for AD were included, regardless of blinding, publication status or language. Reviews, comments, cases, clinical experiences or trials were excluded.

#### Types of experimental animals

Animal models of AD were included, regardless of animal species, gender, age and methods of model establishment. Models of other kinds of dementia, such as vascular dementia or Parkinson’s disease, were excluded.

#### Types of intervention and comparator

Intervention versus comparator was as follows: A, ECC versus non-functional liquid/normal saline/no treatment; B, ECC versus western conventional medicine (WCM); C, ECC plus WCM versus WCM. ECC included CDPS, GCs and PhGs such as ECH, AS and tubuloside B, regardless of dose, form, administration method, or duration. However, ECC plus acupuncture/other CHMs versus acupuncture/other CHMs were excluded.

#### Types of outcome measures

The primary outcome measures were neurobehavioral function indices (NFIs) such as the Morris water maze test and step-down test. The secondary outcome measures were neurobiochemical and neuropathologic changes.

### Data extraction

Two independent authors extracted data from the qualified articles according to a standardized data extraction form. The data of the highest dose were included when the treatment groups included various doses of the drug. The result of the peak time point was included when the data were expressed at different times. If published outcome data were demonstrated graphically, we made an effort to contact the author for further information. Digital ruler software was applied when a response was not received.

### Risk of bias in individual studies

The risk of bias was assessed by the nine-item scale [30] and our previous publications [31] with minor modifications. In Item G, we considered the involvement of aged or female animals. Each item was given one point. Two reviewers independently evaluated the study quality. Divergences were well settled through consulting with correspondence authors.

**Figure 1.**
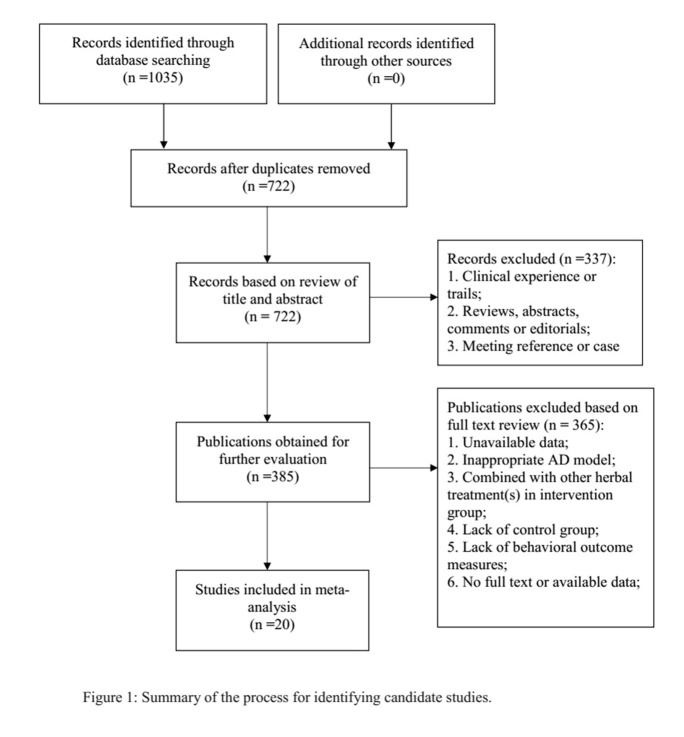
Summary of the process for identifying candidate studies.

### Statistical analysis

The pooled analyses were carried out using RevMan 5.3 software. Heterogeneity across the subgroups was assessed using the Cochrane Q-statistic test and the *I^2^* statistic test. A fixed effects model (*I^2^*< 50%) or a random effects model (*I^2^*> 50%) was used depending on the value of *I^2^*. We calculated the standard mean difference (SMD) with 95% confidence intervals (CIs). Sensitivity analyses omitting one study at a time from the original analysis were conducted to demonstrate our main results to be robust. Considering two-tailed statistical tests, results were considered statistically significant when *P*< 0.05.

## RESULTS

### Study selection

A total of 1035 hits were found through the electrical database searching, of which 313 studies were duplicated. After screening titles and abstracts, 337 studies were excluded because they were clinical trials, case reports or review articles. Through full-text evaluation of the remaining 385 studies, 365 were excluded for at least one of the following reasons: (1) unavailable data; (2) inappropriate AD model; (3) combined with other herbal treatment(s) in the intervention group; (4) no control group; (5) no behavioral outcome measures. Eventually, 20 studies [32-51] were selected; see Fig. 1.

**Figure 2.**
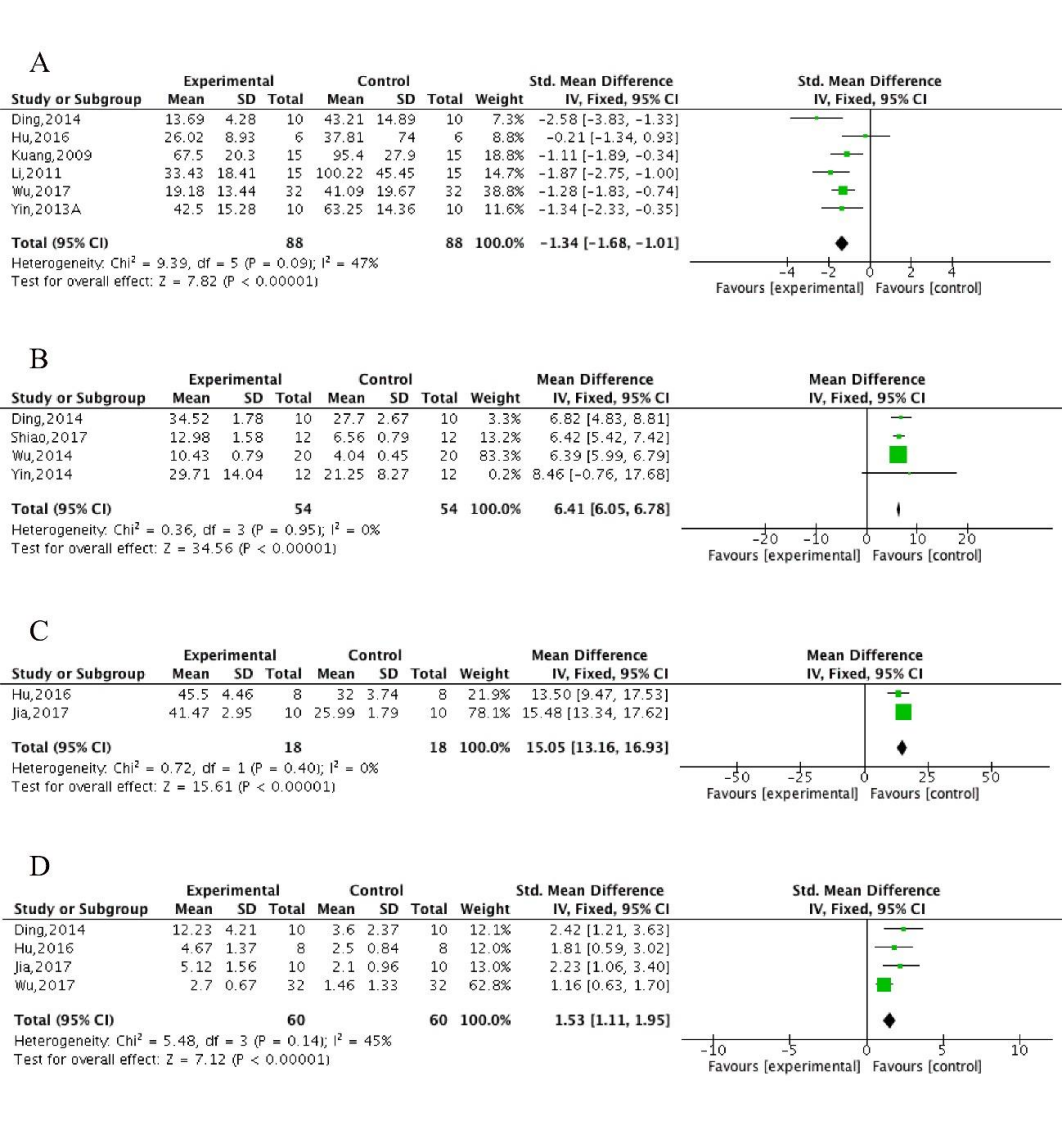
The forest plot in Morris water maze test. Effects of ECC for (A) decreasing the escape latency in spatial performance, increasing (B) exact time/(C) percentage of time and (D) increasing crossing numbers in platform-quadrant in probe test compared with control group.

### Characteristics of included studies

All studies were conducted in China and published between 2001 and 2017, of which 4 studies were published in English [33, 42, 45, 49]. In total, 1696 rats or mice were involved, and the sample size ranged from 40 to 192. A total of 6 different experimental rodent species were involved, including Kunming (KM) mouse (n = 1016, 59.91%), senescence-accelerated mouseprone 8 (SAMP8) mouse (n = 80, 4.72%), NIH mouse (n = 60, 3.54%), amyloid precursor protein/presenilin 1 (APP/PS1) transgenic mouse (n = 40, 2.36%), Sprague-Dawley (SD) rat (n = 440, 25.94%) and Wistar rat (n = 60, 3.54%). Twelve studies used male rodents, 6 studies used both female and male rodents and the other 2 studies did not provide gender details. GCs were used in 13 studies, CDPS in 5 studies, AS in 4 studies, ECH in 2 studies and PhGs in 1 study. AD models were established by using Aβ(1-42), Aβ(25-35) or Aβ(1-40) cerebral ventricle infusion (n = 7), using D-galactose (D-gal, n = 6), scopolamine (n = 6), sodium nitrite (n = 4), aluminium chloride (AlCl_3_, n = 2) or quinolinic acid (n = 1) intraperitoneal injection, or using SAMP8 mice (n = 2) and APP/PS1 transgenic mice (n = 1) directly. The non-functional liquid/normal saline/no treatment control was introduced in all 20 studies; however, WCM control was introduced in 6studies, by donepezil [33, 47, 48] or huperzine A [40, 50, 51]. NFIs as primary outcome measures were carried out by the Morris water maze test (n = 11), step-down test (n = 10), electrical Y-maze test (n = 4), step-through test (n = 3), open field test (n = 2) and passage water maze test (n = 1); see Table 1.

**Table 1:** Characteristics of the included studies.

Study (years)	Type of herbal or bioactive compound	Species Sex Weight N	Anesthetic	Model (method)	Experimental group	Control group	Outcome measure	Intergroup differences*
Kuang, 2009	GCs	KM mice M 18-22g 75	-	D-gal and sodium nitrite	GCs (60, 120 mg/kg) ig, 40~50d	NS for same volume	1. Step-down test 1.1.1 error number (T) 1.1.2 wrong react latency (T) 1.2.1 error number (RT) 1.2.2 wrong react latency (RT) 2. Step-through test 2.1.1 error number (T) 2.1.2 latency (T) 2.2.1 error number (RT) 2.2.2 latency (RT) 3. Morris water maze test escape latency	1.1.1 P<0.05 1.1.2 P>0.05 1.2.1 P<0.05 1.2.2 P>0.05 2.1.1 P<0.05 2.1.2 P>0.05 2.2.1 P<0.05 2.2.2 P<0.05 3. P<0.01
		KM mice M 18-22g 50	-	D-gal and sodium nitrite	GCs (60, 120 mg/kg) ig, 40~50d	NS for same volume	4. Na+-K+ ATPase 5. GSH-PX	4. P<0.05 5. P<0.001
		SD rat M 180-200g 40	-	D-gal and sodium nitrite	GCs (60, 120 mg/kg) ig, 40~50d	NS for same volume	6. SOD 7. NO	6. P<0.001 7. P<0.01
Wu, 2014	GCs	SD rats M 300-350g 100	phenobarbital	Aβ (1-42)	GCs (100, 200 mg/kg) ig, 7-14d	a. sterile distilled water for same volume b. donepezil (0.75 mg/kg)	1.Open field test 1.1 time spend in the hole 1.2 number of entries 1.3 movement activity 2.Step-through test latency (T) 3. Morris water maze test 3.1 escape time 3.2 exact time in platform-quadrant 3.3 swimming velocity 4. Aβ (1-42) deposition 5. Neurotransmitters and metabolites (ACh, NE, DA) 6. Activity of AChE, MAO-A and MAO-B	1.1 P>0.05 1.2 P>0.05 1.3 P>0.05 2. P<0.001 3.1 P<0.05 3.2 P<0.001 3.3 P>0.05 4. P<0.01 5. P<0.05 6. P<0.05
Liu, 2005	GCs	KM mice M 20-24g 60	chloral hydrate	Quinolinic acid	GCs (62.5, 125, 250 mg/kg) ig, 15d	sterile distilled water for same volume	1.Step-down test 1.1 error number (T) 1.2 error number (RT) 2. Electrical Y- maze test right react times 3. Activity of SOD, MDA and GSH-PX. 4. Neuron apoptosis 5. Calcium content	P<0.05 P<0.05 2. P<0.05 3. P P<0.05 or P<0.01 4. P<0.01 5. NG
Liu, 2006	GCs	NIH mice M 20-24g 60	chloral hydrate	Aβ (25-35)	GCs (62.5, 125, 250 mg/kg) ig, 17d	sterile distilled water for same volume	1. Step-down test 1.1 error number (T) 1.2 error number (RT) 2. Activity of SOD, MDA and GSH-PX 3. Neuron apoptosis 4. Bax / Bcl-2	P<0.01 P<0.01 2. P<0.05 or P<0.01 3. P<0.01 4. NG
Luo, 2007	GCs	KM mice M 20-24g 60	chloral hydrate	AlCl3	GCs (62.5, 125, 250 mg/kg) ig, 20d	NS for same volume	1. Step-down test 1.1 error number (T) 1.2 wrong react latency (T) 2. Electrical Y-maze test error react times 3. Activity of SOD and MDA 4. Brain weight coefficient	1.1 P<0.05 1.2 P<0.05 2. P<0.01 3. P<0.05 or P<0.01 4. P<0.01
Luo, 2013	GCs	SD rats M 220-270g 60	chloral hydrate	Aβ (25-35)	GCs (40, 80, 120 mg/kg) ig, 14d	NS for same volume	1. Step-down test 1.1 error number (T) 1.2 reaction time (T) 2. Electrical Y-maze test error react times 3. Activity of AchE 4. Calcium content	1.1 P<0.01 1.2 P<0.01 2. P<0.01 3. P<0.01 4. NG
Yin, 2013(A)	CDPS	SD rats M/F 200-250g 60	chloral hydrate	Aβ (25-35)	CDPS (20, 40, 80 mg/kg) ig, 28d	NS for same volume	1. Morris water maze test escape latency 2. Neuron apoptosis 3. Expression of Bcl-2 and caspase-3	1. P<0.01 2. P<0.01 3. P<0.01
Yin, 2013(B)	CDPS	Wistar rats NG 180-220g 60	chloral hydrate	Aβ (1-40)	CDPS (L, M, H) ig, 28d	corn oil for same volume	1. Morris water maze test escape latency 2. Activity of SOD and MDA 3. Activity of NO, ONOO- and ROS	1. P<0.01 2. P P<0.05 or P<0.01 3. P<0.05 or P<0.01
Li, 2011	CDPS	KM mice M/F 18-22g 75	-	Scopolamine	CDPS (10, 20, 60 mg/kg) NG	NS for same volume	1. Passage water maze test 1.1 error number (T) 1.2 latency (T) 2. Morris water maze test escape latency 3. Activity of SOD, MDA and AChE	P<0.01 P<0.01 2. P<0.05 3. P<0.05 or P<0.01
Ding, 2014	ECH	SD rats M 290-320g 60	chloral hydrate	D-gal and Aβ (25-35)	ECH (10, 20, 40 mg/kg) ig, 28d	a. NS for same volume b. huperzine-A (0.02 mg/kg)	1. Morris water maze test 1.1 escape latency 1.2 number of platform crossing 1.3 exact time in platform-quadrant 2. Activity of NE, DA and 5-TH	1.1 P<0.01 1.2 P<0.01 1.3 NG 2. P<0.05
Peng, 2014	AS	KM mice F 16-20g 120	-	D-gal and AlCl3	AS (30, 60, 120 mg/kg) ig, 30d	NS (10 ml/kg)	1. Step-down test 1.1.1 error number (T) 1.1.2 wrong react latency (T) 1.2.1 error number (RT) 1.2.2 wrong react latency (RT) 2. Level of NO 3.Pathomorphological changes in the hippocampus 4. Expression of Caspase-3	1.1.1 P<0.01 1.1.2 P>0.05 1.2.1 P<0.01 1.2.2 P>0.01 2. P<0.01 3. NG 4. P<0.05
Hu, 2016	AS	APP/PSI mice NG 25-35g 40	-	-	AS (30, 60, 120 mg/kg) ig, 60d	sterile distilled water for same volume	1.Morris water maze test 1.1 escape latency 1.2 number of platform crossing 1.3 percentage of time in platform-quadrant 2. Neuron apoptosis 3. Survival neuron number 4. Aβ (1-42) deposition	1. 1 P<0.01 1.2 P<0.05 1.3 P<0.01 2. P<0.05 3. P<0.05 4. P<0.05
Jia, 2014	GCs	10-month-old SAMP8 mice M 25-35g 40	-	-	GCs (100 mg) ig, 30d	NS for same volume	1. Morris water maze test 1.1 escape latency 1.2 number of platform crossing 1.3 time in the target quadrant 1.4 swimming speed 2. Survival neuron number 3. Activity of MDA, SOD and GSH-PX	1. 1 P<0.01 1.2 P<0.01 1.3 P<0.01 1.4 P>0.05 2. P<0.01 3. P<0.05 or P<0.01
Jia, 2017	PhG	10-month-old SAMP8 mice M 30g 40	-	-	PhG (25, 50, 100 mg/kg) ig, 30d	NS for same volume	1. Morris water maze test 1.1 escape latency 1.2 number of platform crossing 1.3 percentage of time in platform-quadrant 1.4 path length 2. Activity of MDA, SOD and GSH-PX 3.Density of dendritic spines 4.Expression of SYN and PSD-95	P<0.05 P<0.05 P<0.05 P<0.05 2. P<0.05 or P<0.01 3. P<0.05 4. P<0.05
Gao, 2005	GCs	KM mice M/F 18-22g 180	-	Scopolamine	GCs (L, M, H) ig, 30d	sterile distilled water for same volume	1. Step-down test 1.1 error number (T) 1.2 wrong react latency (T)	P<0.01 1.2 P<0.01
Wu, 2017	CDPS	KM mice M/F 18-22g 192	-	D-gal	CDPS (25, 50, 100 mg/kg) ig, 42d	NS for same volume	1. Morris water maze test 1.1 escape latency 1.2 number of platform crossing	P<0.05 P<0.05
Yin, 2014	CDPS	KM mice M/F 23-27g 72	-	Scopolamine	CDPS (25, 50, 100 mg/kg) ig, 42d	a. sterile distilled water for same volume b. donepezil (0.8 mg/kg)	Morris water maze test 1.1 escape latency 1.2 exact time in platform-quadrant 1.3 path length 2. Step-down test 2.1.1 error number (T) 2.1.2 right latency (T) 2.2.1 error number (RT) 2.2.2 right latency (RT) 3. Expression of GAP-43 and SYP 4. Number and morphology of synapses	1.1 NG 1.2 P<0.05 1.3 NG 2.1.1 P<0.05 2.1.2 P>0.05 2.2.1 P<0.05 2.2.2 P<0.05 3. P<0.05 4. P<0.05
Shiao, 2017	ECH	SD rat M 300-350 120	phenobarbital	Aβ (1-42)/ Scopolamine	ECH (2.5, 5.0 mg/kg) ig, 15d	a. sterile distilled water for same volume b. donepezil (0.75 mg/kg)	1. Open-field task 1.1 time spend in the hole 1.2 number of entries into the hole 1.3 movement activity 2. Step-through test 2.1 latency (T) 2.2 latency (RT) 3. Morris water maze test 3.1 escape latency 3.2 exact time in platform-quadrant 3.3 swimming velocity 4. Aβ (1-42) deposition 5. Levels of Ach, NE and DA 6. Activity of AChE, MAO-A and MAO -B	1.1 P<0.05 1.2 P<0.05 1.3 P>0.05 2.1 P<0.05 2.2 P>0.05 3.1 P<0.05 3.2 P<0.05 3.3 P>0.05 4. P<0.05 5. P<0.05 or P<0.01 6. P<0.05 or P<0.01
Piao, 2001	AS	KM mice M 18-22 60	-	Scopolamine	AS (5, 10 mg/kg) ig, 10d	a. NS for same volume b. huperzine-A (0.07 mg/kg)	1. Step-down test 1.1.1 wrong react latency (T) 1.1.2 error time (T) 1.2.1 wrong react latency (RT) 1.2.2 error time (RT) 2. Electrical Y-maze test right react times 3. Activity of AChE	1.1.1 NG 1.1.2 P>0.05 1.2.1 P<0.05 1.2.2 P<0.05 2. P<0.05 3. P<0.05
Lin, 2012	AS	KM mice M 18-22g 72	-	Scopolamine	AS (30, 60, 120mg/kg) ig, 10d	a. NS for same volume b. huperzine-A (0.07 mg/kg)	1. Step-down test 1.1.1 error number (T) 1.1.2 wrong react latency (T) 1.2.1 error number (RT) 1.2.2 wrong react latency (RT) 2. Activity of MDA, SOD and GSH-PX 3. Protein content in brain tissue	1.1.1 NG 1.1.2 NG 1.2.1 P<0.01 1.2.2 P<0.01 2. P<0.05 or P<0.01 3. P>0.05

Note. GCs: glycosides of Cictanches. CDPS: polysacchrides of Cistanches deserticola. ECH: echinacoside. AS: acteoside. PhGs: phenylethanoid glycosides. KM mice: Kunming mice. SD rats: Sprague-Dawley rats. NIH mice: National Institutes of Health mice. SAMP8 mice: senescence-accelerated mouseprone 8 mice. APP/PS1 mice: amyloid precursor protein/presenilin 1 transgenic mice. M: male. F: female. -: no. D-gal: D-galactose. NG, not given. Aβ: amyloid β. AlCl_3_: aluminium chloride. ig: intragastric administration. L: low dose. M: medium dose. H: high dose. d: day. NS: normal saline. T: in the training test. RT: in the retention test. Na^+^-K^+^ ATPase: sodium-potassium adenosine triphosphatase. GSH-P_X_ : glutathione peroxidase. SOD: superoxide dismutase. NO: nitric oxide. AChE: acetylcholinesterase. ACh: acetylcholine. NE: norepinephrine. DA: dopamine. MDA: malondialdehyde. 5-TH: 5-hydroxytryptamine. MAO-A: monoamine oxidase A. MAO-B: monoamine oxidase B. Bax: B-cell lymphoma/leukemia-2 associated X protein. Bcl-2: B-cell lymphoma/leukemia-2.

* the intergroup differences listed were ECC vs. negative control group (NS or sterile distilled water) , the intergroup differences of ECC vs. modern western conventional treatments were not given.

**Figure 3.**
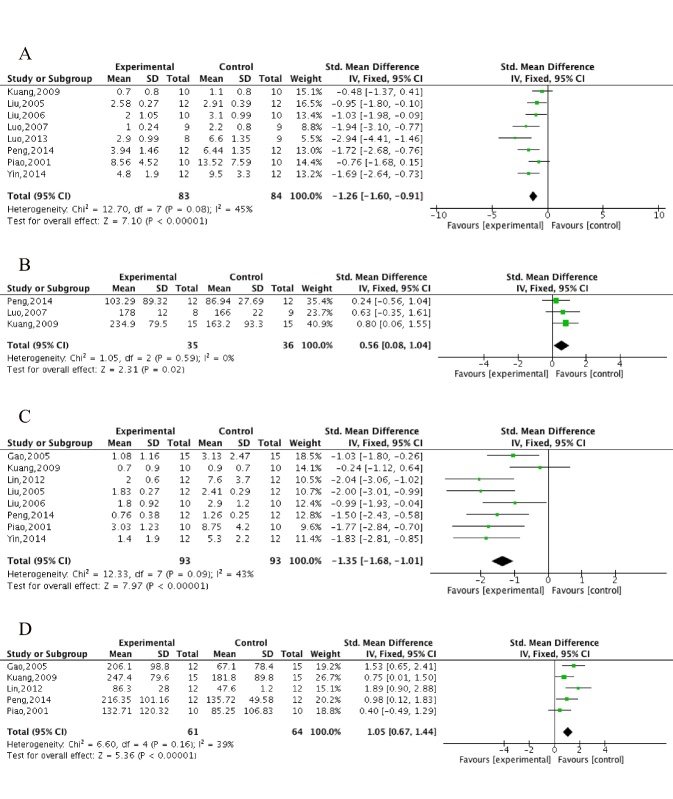
The forest plot in Step-down test. Effects of ECC for decreasing (A) error times and (B) wrong react latency in training test and decreasing (C) error times and (D) wrong react latency in retention test compared with control group.

### Study quality

The quality of the 20 included studies ranged from 4 to 7, with a mean score of 5.05. All studies used random allocation and declared no potential conflict of interests. Nineteen studies were peer reviewed, while one study [32] was an online PhD thesis. The use of anesthetic without significant intrinsic neuroprotective activity was reported in 18 studies, compliance with animal welfare regulations in 10 studies, control of temperature in 8 studies and animal models with relevant comorbidities in 8 studies. No study reported sample size calculation or blinded assessment of the model or outcome; see Table 2.

**Figure 4.**
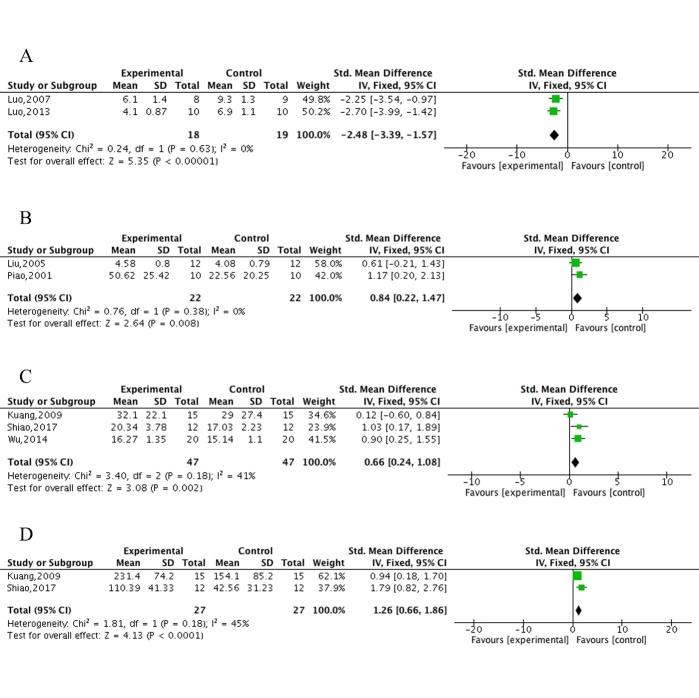
The forest plot in Electrical Y-maze test and Step-through test. Effects of ECC for (A) decreasing error react times, (B) increasing right react times in Electrical Y-maze test, and decreasing latency in training test (C) / retention test (D) in Step-through test compared with control group.

### Effectiveness

#### Neurobehavioral function indices

The Morris water maze test, including the spatial test and the probe test, was conducted in 11 studies [32, 33, 38-41, 43, 45, 47-49]. All 11 studies reported the spatial test using the escape latency as an outcome measure, of which 3 studies [33, 48, 49] provided graphic data, and we failed to apply digital ruler software or to get in touch with the author for further information. Meta-analysis of 8 studies showed ECC significantly decreased the escape latency compared with the control (n = 216, MD = -1.46, 95%CI [-1.79 to -1.12], *P*<0.00001; heterogeneity: χ^2^ = 58.87, df = 7 (*P*<0.00001); *I*^2^ = 88%). Owing to obvious heterogeneity, we used sensitivity analyses and removed the respective outlier studies. Meta-analysis of 6 studies [32, 38, 40, 41, 43, 47] showed a significant effect of ECC in decreasing the escape latency in spatial performance compared with control (n = 176, MD = -1.34, 95%CI [-1.68 to -1.01], *P*<0.00001; heterogeneity: χ^2^ = 9.39, df = 5 (*P* = 0.09); *I*^2^ = 47%; Fig. 2A). Four studies [33, 41, 48, 49] showed an insignificant decrease in escape latency in ECC group compared with WCM control; however, meta-analysis was failed owing to unavailable data in 3 studies [33, 48, 49]. In the probe test, meta-analysis of 4 studies [33, 41, 48, 49] showed ECC were significant for increasing exact time in platform-quadrant (n = 108, MD = 6.41,95%CI [6.05 to 6.78], *P*<0.00001; heterogeneity: χ^2^ = 0.36, df = 3 (*P* = 0.95); *I*^2^ = 0%; Fig. 2B), 2 studies [43, 45] for increasing percentage of time in the platform-quadrant (n = 36, MD = 15.05, 95%CI [13.16 to 16.93], *P*<0.00001; heterogeneity: χ^2^ = 0.72, df = 1 (*P*= 0.400); *I*^2^ = 0%; Fig. 2C) and 4 studies [41, 43, 45, 47] for increasing number of platform crossings (n = 120, MD = 1.53, 95%CI [1.11 to 1.95], *P*<0.00001; heterogeneity: χ^2^ = 5.48, df = 3 (*P* = 0.14); *I*^2^ = 45%; Fig. 2D) compared with controls. Meta-analysis of 4 studies [33, 41, 48, 49] showed there were no significant intergroup differences between the ECC group and WCM controls in increasing the exact time in the platform-quadrant (n = 108, MD = 0.81,95%CI [0.35 to 1.27], *P* = 0.06; heterogeneity: χ^2^ = 18.62, df = 3 (*P* = 0.03); *I*^2^ = 84%). We carried out a sensitive analysis by removing one study [49] of an obviously less effective ECC dose and markedly reduced the heterogeneity (n = 84, MD = 0.46, 95%CI [-0.03 to 0.94], *P* = 0.07; heterogeneity: χ^2^ = 0.94, df = 3 (*P* = 0.63); *I*^2^ = 0%; Supplementary Fig. 1A). Two studies [45, 48] showed ECC significantly decreased the total swimming length (*P*<0.05), whereas two other studies [33, 49] showed there were no significant difference in reducing the swimming velocity (*P*>0.05) compared with controls.

**Figure 5.**
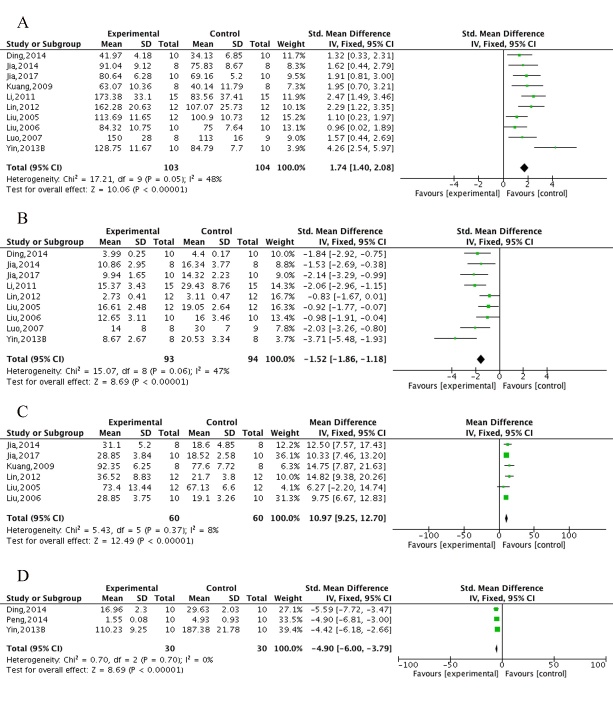
The forest plot of oxidative stress. Effects of ECC for increasing the activity of (A) SOD and (C) GSH-Px, decreasing (B) MDA and (D) NO compared with control group.

**Figure 6.**
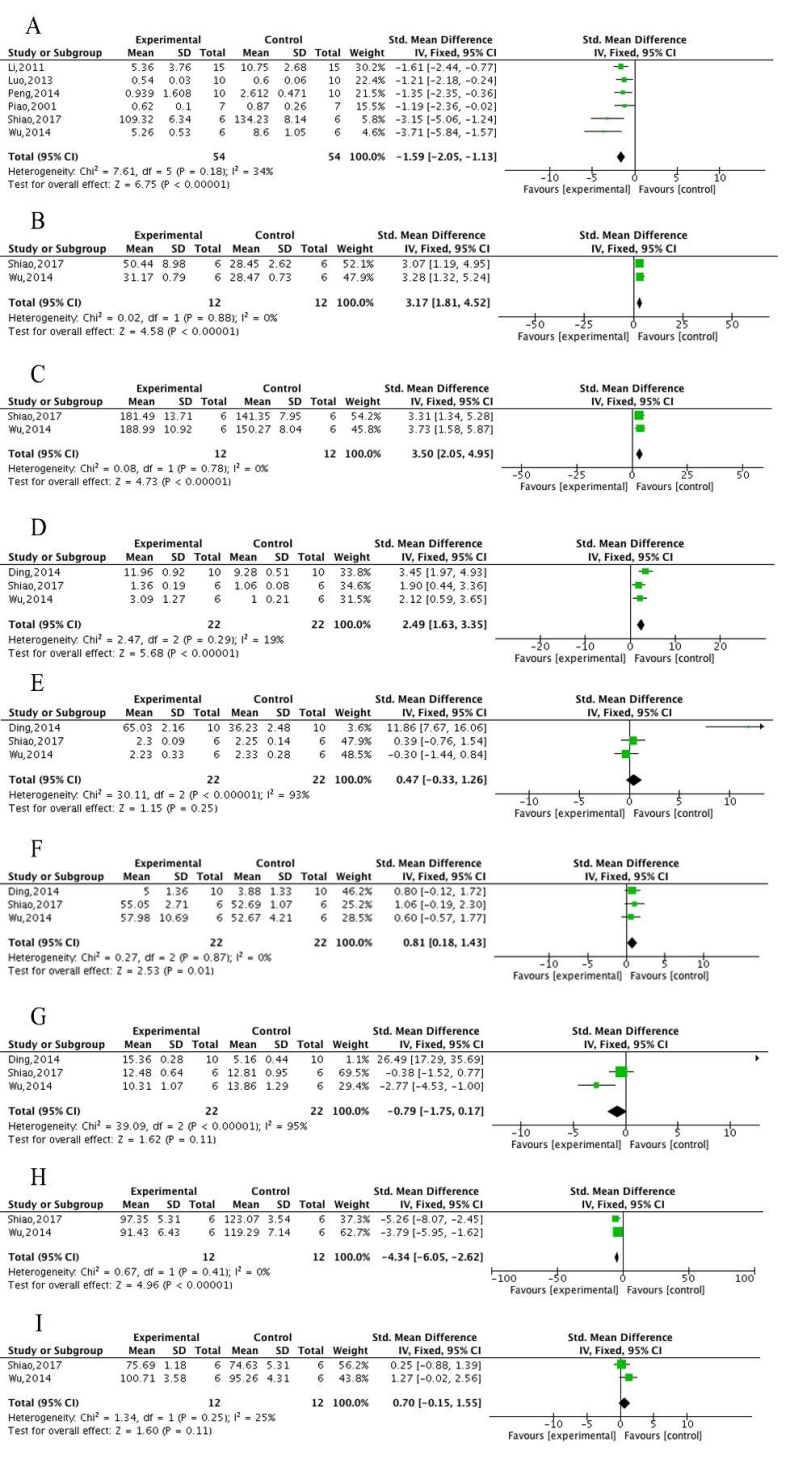
The forest plot of AChE and neurotransmitters. Effects of ECC for (A) decreasing the activity of AChE, increasing the level of Ach in hippocampus (B)/in cortex (C), increasing the level of DA in hippocampus (D)/in cortex (E), increasing the level of NE in hippocampus (F)/in cortex (G), and decreasing the activity of MAO-A in hippocampus (I)/in cortex (H) compared with control group.

**Table 2:** Risk of bias of the included studies.

Study	A	B	C	D	E	F	G	H	I	Total
Kuang, 2009	-	+	+	-	+	-	-	+	+	5
Wu, 2014	+	+	+	-	-	-	-	+	+	5
Liu, 2005	+	-	+	-	+	-	-	-	+	4
Liu, 2006	+	-	+	-	+	-	-	-	+	4
Luo, 2007	+	-	+	-	+	-	-	-	+	4
Luo, 2013	+	-	-	-	+	-	-	-	+	3
Yin, 2013 (A)	+	-	+	-	+	+	-	-	+	5
Yin, 2013 (B)	+	-	+	-	+	?	-	-	+	3
Li, 2011	+	-	+	-	+	+	-	+	+	6
Ding, 2014	+	+	+	-	+	-	-	+	+	6
Peng, 2015	+	+	+	-	+	+	-	+	+	7
Hu, 2016	+	+	+	-	+	?	-	+	+	6
Jia, 2014	+	+	+	-	+	+	-	+	+	7
Jia, 2017	+	+	+	-	+	+	-	+	+	7
Gao, 2005	+	-	+	-	+	+	-	-	+	5
Wu, 2017	+	-	+	-	+	+	-	+	+	6
Yin, 2014	+	-	+	-	+	+	-	-	+	5
Shiao, 2017	+	+	+	-	-	-	-	+	+	5
Piao, 2001	+	-	+	-	+	-	-	-	+	4
Lin, 2012	+	-	+	-	+	-	-	-	+	4

*Note*. Studies fulfilling the criteria of A: peer reviewed publication; B: control of temperature; C: random allocation to treatment or control; D: blinded induction of model or outcome; E: use of anesthetic without significant intrinsic neuroprotective activity; F: animal model (aged or female involved); G: sample size calculation; H: compliance with animal welfare regulations; I: statement of potential conflict of interests. + = Yes, - = No, ? = unclear.

The step-down test, including the training test for learning score and retention test for memory score, was conducted in 10 studies [32, 34-37, 42, 46, 48, 50, 51]. Meta-analysis of 8 studies [32, 34-36, 37, 42, 48, 50] showed ECC were significant for decreasing the error times (n = 167, MD = -1.26, 95%CI [-1.60 to -0.91], *P*<0.00001; heterogeneity: χ^2^ = 12.70, df = 7 (*P* = 0.08); *I*^2^ = 45%; Fig. 3A), 3 studies [32, 36, 42] for decreasing wrong reaction latency (n = 71, MD = 0.56, 95%CI [0.08 to 1.04], *P* = 0.02; heterogeneity: χ^2^ = 1.05, df = 2 (*P* = 0.59); *I*^2^ = 0%; Fig. 3B), 2studies [37, 48] for increasing right reaction latency (*P*<0.05), 8 studies [32, 34, 35, 42, 46, 48, 50, 51] for decreasing error times in the retention test (n = 186, MD = -1.35, 95%CI [-1.68 to -1.01], *P*<0.00001; heterogeneity: χ^2^ = 12.33, df = 7 (*P* = 0.09); *I*^2^ = 43%; Fig. 3C), 5 studies [32, 42, 46, 50, 51] for decreasing wrong reaction latency in the retention test(n = 125, MD = 1.05, 95%CI [0.67 to 1.44], *P*<0.00001; heterogeneity: χ^2^ = 6.60, df= 4 (*P* = 0.16); *I*^2^ = 39%; Fig. 3D) and 1 study [48] for increasing right reaction latency in the retention test (*P*<0.05) compared with controls. Meta-analysis of 3 studies [48, 50, 51] showed there were no significant differences in reaction latency (n = 68, MD = -3.02, 95%CI [-23.88 to 17.84], *P* = 0.78; heterogeneity: χ^2^ = 1.43, df= 2 (*P* = 0.49); *I*^2^ = 0%; Supplementary Fig. 1B) and error time decrease (n = 68, MD = -0.19, 95%CI [-0.71 to 0.34], *P* = 0.49; heterogeneity: χ^2^ = 1.71, df= 2 (*P* = 0.43); *I*^2^ = 0%; Supplementary Fig. 1C) in the retention test between the ECC group and WCM controls.

The electrical Y-maze test was conducted in 4 studies [34, 36, 37, 50]. Meta-analysis of 2 studies [36, 37] showed ECC were significant for decreasing error reaction times (n = 37, MD = -2.48, 95%CI [-3.39 to -1.57], *P*<0.00001; heterogeneity: χ^2^ = 0.24, df= 1 (*P* = 0.63); *I*^2^ = 0%; Fig. 4A), 2 studies [34, 50] for increasing right reaction times (n = 44, MD = 0.84, 95%CI [0.22 to 1.47], *P* = 0.008; heterogeneity: χ^2^ = 0.76, df = 1 (*P* = 0.38); *I*^2^ = 0%; Fig. 4B) and 1 study [34] for increasing right reaction times in the retention test (*P*<0.05) compared with controls. The step-through test was performed in 3 studies [32, 33, 49]. Meta-analysis of 3 studies showed ECC were significant for decreasing latency in the training test (n = 94, MD = 0.66, 95% CI [0.24 to 1.08], *P* = 0.002; heterogeneity: χ^2^ = 2.40, df = 2 (*P* = 0.18); *I*^2^ = 41%; Fig. 4C) and in the retention test (n = 54, MD = 1.26, 95% CI [0.66 to 1.86], *P*<0.0001; heterogeneity: χ^2^ = 1.81, df = 1 (*P* = 0.18); *I*^2^ = 45%; Fig. 4D) and 1 study [32] showed ECC significantly decreased the number of errors in both the training test and the retention test (*P*<0.05) compared with controls. The open field test was conducted in 2 studies [33, 49] that both clearly showed ECC increased the frequency of visits and time spent in the hole compared with controls (*P*<0.05), while 1 study [40] indicated that ECC markedly reduced the escape latency and the number of errors (*P*<0.01) compared with controls. However, compared with WCM controls, ECC were statistically less effective in increasing the frequency (n = 64, MD = -3.6, 95%CI [-4.49 to -2.7], *P*<0.001; heterogeneity: χ^2^ = 1.10, df = 1(*P* = 0.29); *I*^2^ = 9%; Supplementary Fig. 1D) and time spent in the hole (n = 64, MD = -3.44, 95%CI [-4.36 to -2.53], *P*<0.001; heterogeneity: χ^2^ = 1.24, df = 1 (*P* = 0.26); *I*^2^ = 20%; Supplementary Fig. 1E).

#### Neuroprotective mechanisms

Compared with controls, meta-analysis of 10 studies [32, 34-36, 39-41, 44, 45, 51] showed significant effects of ECC in increasing the activity of SOD (n = 207, MD = 1.74, 95%CI [1.40 to 2.08], *P*<0.00001; heterogeneity: χ^2^ = 17.21, df = 9 (*P* = 0.05); *I*^2^= 48%; Fig. 5A) and 9 studies [34-36, 39-41, 44, 45, 51] in decreasing MDA (n = 187, MD = -1.52, 95%CI [-1.86 to -1.18], *P*<0.00001; heterogeneity: χ^2^ = 15.07, df = 8 (*P* = 0.06);*I*^2^ = 47%; Fig. 5B). Compared with controls, meta-analysis of 6 studies [32, 34, 35, 44, 45, 51] showed significant effects of ECC in increasing GSH-Px (n = 120, MD = 10.97, 95%CI [9.25 to 12.70], *P*<0.00001; heterogeneity: χ^2^ = 5.43, df = 5 (*P* = 0.37); *I*^2^ = 8%; Fig. 5C) and 3 studies [39, 41, 42] in decreasing NO (n = 60, MD = -4.90, 95%CI [-6.00 to -3.79], *P*<0.00001; heterogeneity: χ^2^ = 0.70, df = 2 (*P* = 0.70); *I*^2^ = 0%; Fig. 5D). Meta-analysis of 2 studies [33, 51] showed there were insignificant differences between ECC groups and WCM controls in increasing the activity of both SOD (n = 44, MD = -2.93, 95%CI [-7.05 to 1.18], *P* = 0.16; heterogeneity: χ^2^=0.62, df = 1 (*P* = 0.43); *I*^2^= 0%; Supplementary Fig. 2A) and MDA (n = 44, MD = -0.17, 95%CI [-0.84 to 0.49], *P* = 0.61; heterogeneity: χ^2^ = 14.57, df = 1 (*P* = 0.0001); *I*^2^= 93%; Supplementary Fig. 2B). One study indicated there were insignificant differences between ECC and WCM groups in both increasing GSH-Px [51] and decreasing NO [41].

**Figure 7.**
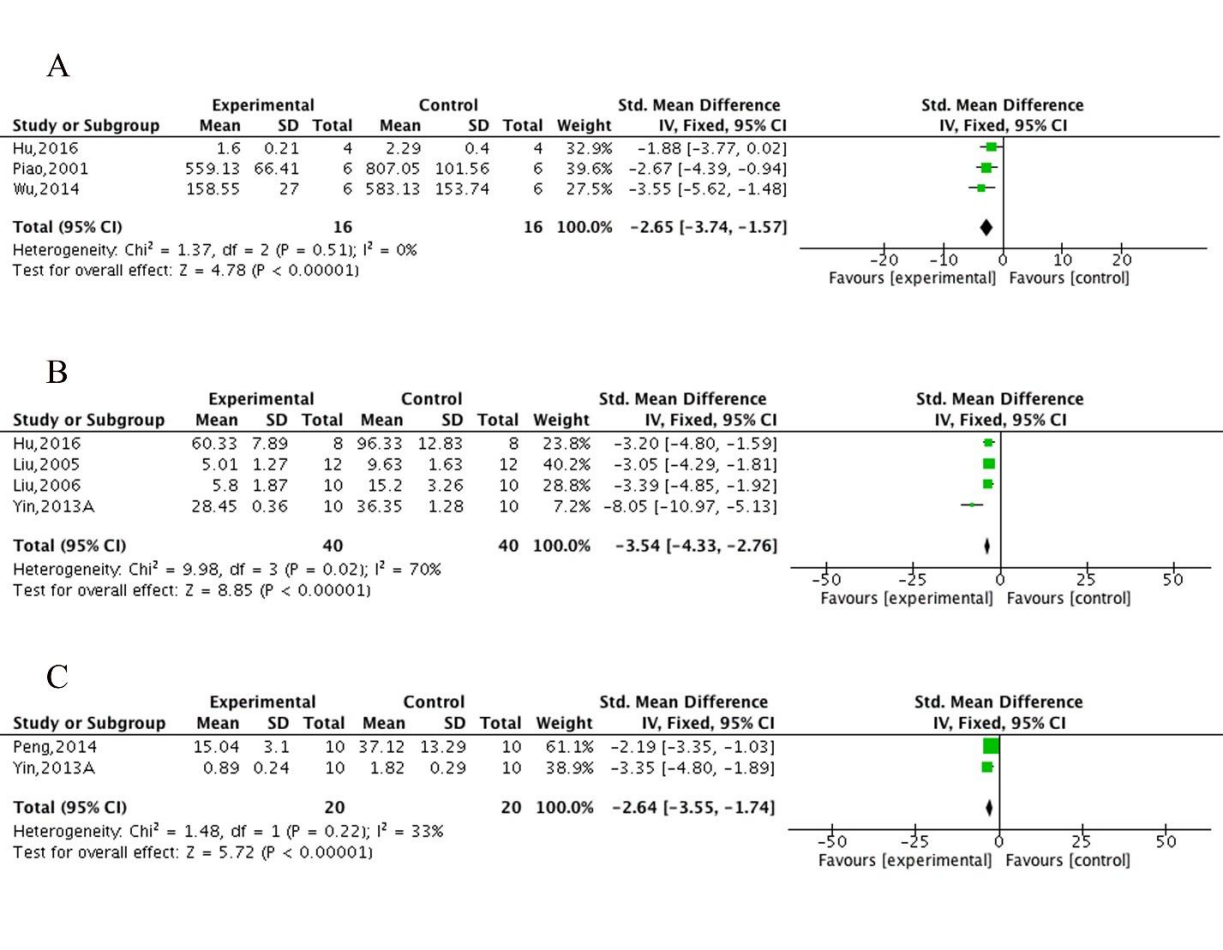
The forest plot of neuropathologic changes and Caspase-3. Effects of ECC for (A) decreasing Aβ deposition, (B) decreasing apoptosis and (C) decreasing Caspase-3compared with control group.

Compared with controls, meta-analysis of 6 studies [33, 37, 40, 42, 49, 50] showed ECC were significant for decreasing the activity of AChE (n = 108, MD = -1.59, 95%CI [-2.05 to -1.13], *P*<0.00001; heterogeneity: χ^2^ = 7.61, df = 5 (*P* = 0.18); *I*^2^ = 34%; Fig. 6A), 2 studies [33, 49] for increasing the level of Ach in both the hippocampus (n = 24, MD = 3.17, 95%CI [1.81 to 4.52], *P*<0.00001; heterogeneity: χ^2^ = 0.02, df = 1 (*P* = 0.88); *I*^2^ = 0%; Fig. 6B) and the cortex (n = 24, MD = 3.50, 95%CI [2.05 to 4.95], *P*<0.00001; heterogeneity: χ^2^ = 0.08, df = 1 (*P* = 0.78); *I*^2^ = 0%; Fig. 6C), 3 studies [33, 41, 49] for increasing the level of DA in the hippocampus (n = 44, MD = 2.49, 95%CI [1.63 to 3.35], *P*<0.00001; heterogeneity: χ^2^ = 2.47, df = 2 (*P* = 0.29); *I*^2^ = 19%; Fig. 6D) but caused an insignificant difference in DA level in the cortex (n = 44, MD = 0.47, 95%CI [-0.33 to 1.26], *P* = 0.25; heterogeneity: χ^2^ = 30.11, df= 2, *P*<0.00001;* I*^2^ = 93%; Fig. 6E), 3 studies [33, 41, 49] for increasing the level of NE in the hippocampus (n = 44, MD = 0.81, 95%CI [0.18 to 1.43], *P* = 0.01; heterogeneity: χ^2^ = 0.27, df = 2 (*P* = 0.87); *I*^2^ = 0%; Fig. 6F) but caused an insignificant difference in NE level in the cortex (n = 44, MD = -0.79, 95%CI [-1.75 to 0.17], *P* = 0.11; heterogeneity: χ^2^ = 39.09, df = 2 (*P*<0.00001); *I*^2^ = 95%; Fig. 6G) and 2 studies [33, 49] in decreasing the activity of monoamine oxidase A (MAO-A) in the cortex (n = 24, MD = -4.34, 95%CI [-6.05 to -2.62], *P*<0.00001; heterogeneity: χ^2^ = 0.67, df= 1 (*P* = 0.41); *I*^2^ = 0%; Fig. 6H) but not in the hippocampus (n = 24, MD = 0.7, 95%CI [-0.15 to 1.55], *P* = 0.11; heterogeneity: χ^2^ = 1.34, df = 1 (*P* = 0.25); *I*^2^ = 25%; Fig. 6I). The change in the activity of monoamine oxidase B (MAO-B) was insignificant in both the cortex and the hippocampus between the ECC group and control group [33, 49]. Compared with WCM controls, meta-analysis of 2 studies [33, 50] showed ECC were insignificant in the activity of AChE (n = 26, MD = 0.09, 95%CI [-0.07 to 0.25], *P* = 0.26; heterogeneity: χ^2^ = 1.54, df = 1 (*P* = 0.22); *I*^2^ = 35%; Supplementary Fig. 2C). Two studies [33, 41] indicated that there was no significant difference between the ECC group and WCM controls in the level of DA (n = 32, MD = 0.14, 95%CI [-0.61 to 0.9], *P* = 0.71; heterogeneity: χ^2^ = 6.84, df = 1 (*P* = 0.009); *I*^2^ = 85%; Supplementary Fig. 2D) and NE (n = 32, MD = -0.77, 95%CI [-2.10 to 0.56], *P* = 0.26; heterogeneity: χ^2^ = 34.22, df = 1 (*P*<0.0001); *I*^2^ = 97%; Supplementary Fig. 2E) in the hippocampus; however, significant differences were found in the level of DA (n = 32, MD = -0.92, 95%CI [-1.36 to -0.48], *P*<0.0001; heterogeneity: χ^2^ = 0.66, df = 1 (*P* = 0.42);* I*^2^ = 0%; Supplementary Fig. 2F) and NE (n = 32, MD = -1.25, 95%CI [-1.53 to -0.98], *P*<0.0001; heterogeneity: χ^2^ = 0.65, df = 1 (*P* = 0.42); *I*^2^ = 0%; Supplementary Fig. 2G) in the cortex. The levels of Ach, MAO-A and MAO-B were given in 1 study [33] and no significant differences were found between the ECC group and WCM controls.

**Figure 8.**
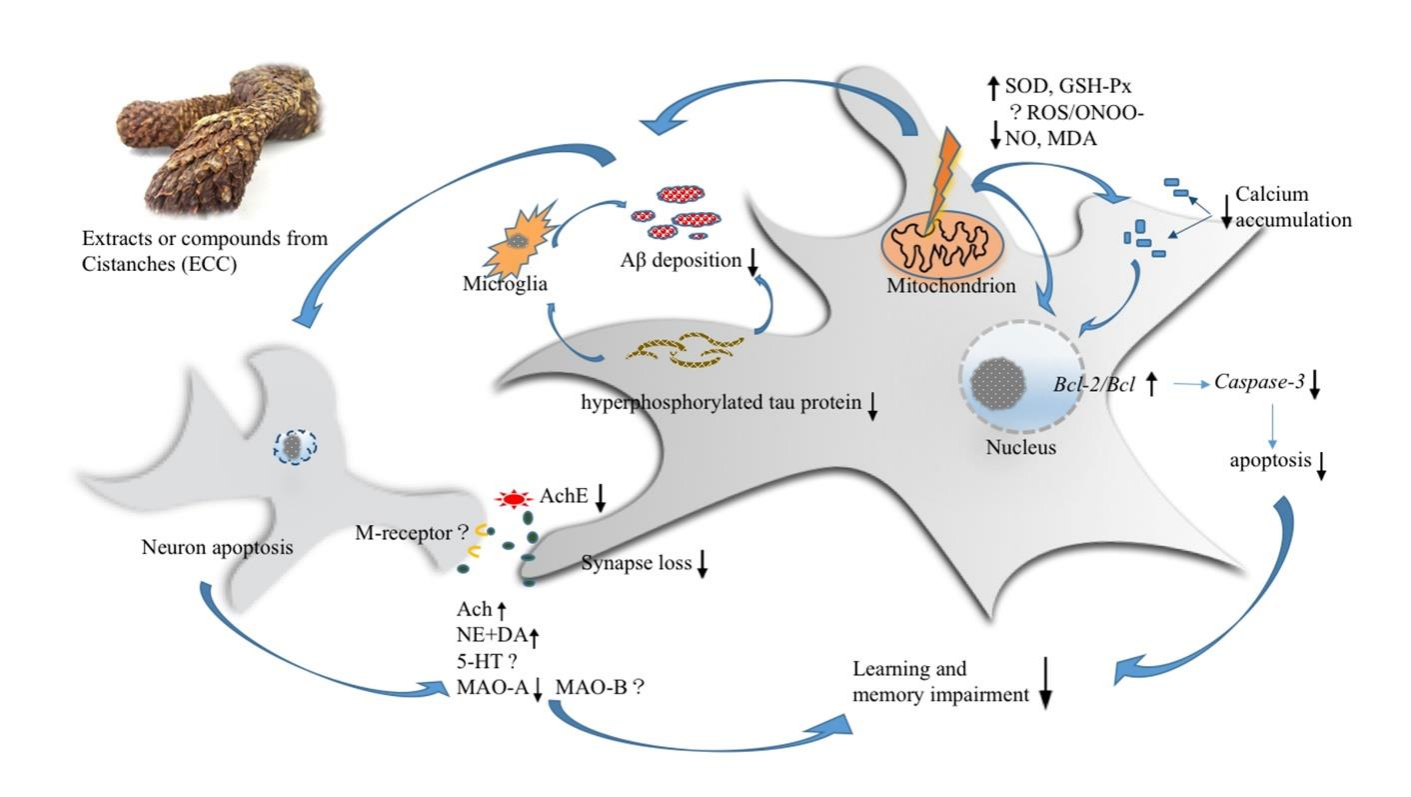
Summary of the possible neuroprotective mechanism of ECC for AD. ECC reduced the excessive ROS in mitochondrion, increased the activity of GSH-PX, SOD, and decreased the level NO and MDA. ECC decreased the level NO, down-regulated the over activation of microglia, exerting potential inhibitory effects on microglia-involved neuro-inflammation. ECC decreased Aβ deposition and tau protein hyper-phosphorylation. ECC decreased the activity of AchE and maintained the normal level of Ach and NE in Cholinergic neuron and increased the level of DA in hippocampus. ECC activated the NMDA -receptor and ameliorated the loss of synapses. The evidence of ECC in increasing the level of 5-HT is inadequate currently. ECC regulate the calcium deposition and maintain neuronal calcium homeostasis. ECC up-regulate the expressions of Bcl-2, decrease the ratio of Bax / Bcl2, down-regulate the expressions of Caspase-3 and reduce neurocyte apoptosis.

Three studies [33, 43, 49] showed ECC were marked in decreasing Aβ deposition (n = 32, MD = -2.65, 95%CI [-3.74 to -1.57], *P*<0.00001; heterogeneity: χ^2^ = 1.37, df = 2 (*P* = 0.51); *I*^2^ = 0%; Fig. 7A), 4 studies [34, 35, 38, 43] in decreasing apoptosis (n = 80, MD = -3.54, 95%CI [-4.33 to -2.76], *P*<0.00001; heterogeneity: χ^2^ = 9.98, df = 3 (*P* = 0.02); *I*^2^ = 70%; Fig. 7B) and 2 studies [38, 42] in decreasing *Caspase-3* (n = 40, MD = -2.64, 95%CI [-3.55 to -1.74], *P*<0.00001; heterogeneity: χ^2^ = 1.48, df= 1 (*P* = 0.22); *I*^2^ = 33%; Fig. 7C). Two studies [34, 37] in decreasing of calcium deposition in the ECC group, with 1 study [37] indicating an increase in synapse number rather than a change in extrinsic features. Two studies [35, 38] obviously showed ECC increased the expression of *Bcl-2* compared with controls. One study [33] provided data of the ECC and WCM control groups on Aβ deposition, but no intergroup differences were found. To summarize, we present a schematic overview of the neuroprotective mechanisms of ECC in AD (Fig. 8).

## DISCUSSION

### Summary of evidence

This is the first preclinical systematic review to assess the efficacy of ECC for experimental AD. Twenty studies with 1696 rodents were selected. The quality of the studies included ranged from 4 to 7. The evidence available from the present study showed that ECC improved cognitive function in experimental AD mainly through mechanisms involving antioxidant stress and antiapoptosic effects, inhibiting Aβ deposition and tau protein hyperphosphorylation and promoting synapse protection.

### Limitations

First, we only searched English and Chinese studies, which may lead to a certain degree of selective reporting and publication bias. It is well known that negative findings are less likely to be published. In the present analysis, some primary studies didn’t provide original data, and some information was inaccessible. Thus, the dominance of positive studies might lead to the efficacy of ECC being overestimated. Second, the study quality was considered moderate, ranging from 4 to 7 out of 9 with a mean score of 5.05, indicating that the results should be explained with caution. Third, heterogeneity may exist due to the variety of AD model selection and preparation. Also, people with AD are always of old age, and a gender difference is observed in AD prevalence. In the present analysis, most AD models used healthy male rodents, which may lead to some challenges in clinical application.

### Implications for practice

Preclinical animal research is the foundation of understanding of human diseases [52-54]; however, original preclinical research is often conducted with a small sample size, leading to less solid conclusions and poor repeatability [55]. The systematic review can integrate comprehensive preclinical evidence efficiently and guide potential clinical translation [56, 57]. The present study showed ECC could improve cognitive function and exert potential neuroprotective effects in experimental AD according to a large amount of experimental animal data, with 1696 rodents, indicating that ECC are candidates for AD treatment and can be used for further clinical trials. Besides, systematic review of animal researches is a more economical and ethical method to integrate preclinical evidence, helping to reduce unnecessary sacrifice of laboratory animals and preventing invalid or less informative researches [58, 59]. Systematic review of preclinical researches can identify defects in study design and implementation, contributing to improvement of the quality of follow-up preclinical researches [60, 61]. In the present analysis, the quality of included studies ranged from 4 to 7 out of 9 points. The main flaws were a lack of sample size calculation, poor blinding in model induction and outcome assessment and an establishment of AD models based on no comorbidities. Reporting guidelines, such as ARRIVE, can provide guidance on the complete and transparent reporting of *in vivo* animal researches regularly and scientifically, helping to improve the quality of further researches [62-65]. Thus, we suggest that further animal researches of AD should follow up the reporting guidelines, increasing the value of clinical trials and further application.

Animal models are essential for understanding the induction and pathogenesis of a disease and developing therapeutic strategies that limit disease progression and eventually lead to effective treatments for the disease [66, 67]. An ideal AD model is essential for preclinical research and should include the following points: (1) correspondence to AD pathogenesis; (2) stable cognitive impairment; (3) low mortality; (4) simple to operate [68, 69]. In the present study, various kinds of AD models were used, including Aβ cerebral ventricle infusion, D-gal, scopolamine, sodium nitrite, AlCl_3_ or quinolinic acid intraperitoneal injection, and using SAMP8 mice and APP/PS1 transgenic mice. The former two kinds of AD models are more cost-effective and accessible, and are widely used in experimental AD research currently [70]; however, they can only partly simulate the pathological features and memory impairment symptoms of AD. In addition, injection injury and ischemia or anoxia in multiple local organs is inevitable. Based on the aging comorbidity and pathology of AD, SAMP8 mice and APP/PSI double transgenic rats are better to mimic the characteristics of AD [71]; however, the weaknesses are a longer preparation time and a higher cost, which to a great extent limits their current use in practice. Further research on ideal AD model is urgently needed, which may also be of great importance in data analysis and preclinical evidence assessment.

The present study demonstrated ECC had neuroprotective effects in AD models according to the neurobehavioral, neurobiochemical and neuro-pathological observations. The mechanisms of ECC for AD are summarized as follows: (1) Antioxidant stress: ECC passed through the injured membrane, affecting the signal pathway of reactive oxygen species (ROS). ECC reduced the amount of excessive ROS in the mitochondrion, increased the activity of GSH-P_X_, SOD and sodium-potassium adenosine triphosphatase (NA^+^-K^+^ATPase) and decreased NO and MDA levels [39, 72, 73]. (2) Regulation of neuroinflammation: ECC decreased the level NO and down-regulated the over-activation of microglia, exerting potential inhibitory effects on microglia involving neuroinflammation [74, 75]. In the included studies, the effects on neuroinflammation of ECC in AD are less pronounced, indicating modification in further research is needed. (3) Resisting Aβ deposition and tau protein hyperphosphorylation: ECC decreased Aβ deposition and tau protein hyperphosphorylation [33, 43, 49], which may have an effect on oxidant stress and neuroinflammation; however, evidence on how ECC inhibit Aβ deposition and tau protein hyper-phosphorylation is lacking, and further preclinical researches *in vitro* are essential. (4) Synapse protection: ECC decreased the activity of AchE, maintained normal Ach and NE levels in cholinergic neurons and increased the level of DA in the hippocampus. ECC activated the NMDA receptor and ameliorated the loss of synapses [48, 50], helping to regulate the proper function of synapses and guarantee essential intercellular contacts. However, evidence on the influence of ECC in increasing the level of 5-hydroxytryptamine (5-HT) in the brain is inadequate currently [41]. (5) Antiapoptosis: ECC can maintain the mitochondrial membrane potential and reduce the amount of excessive ROS, inhibiting the initiation of neural apoptosis [32, 76]. ECC can up-regulate the expression of Bcl-2, decrease the ratio of Bax/Bcl2, down-regulate the expression levels of Caspase-3, P53, P65, SYN, PSD-95 and iNOS [45, 77] and reduce neuron apoptosis eventually. (6) Maintaining neuronal calcium homeostasis: An abnormal calcium steady state is the final common pathway of neuron destruction and is connected to oxidant stress, neuroinflammation, Aβ deposition and tau protein hyperphosphorylation [78, 79]. ECC can protect the neuronal membrane, regulate the opening of calcium channels and maintain neuronal calcium homeostasis [34, 37, 80]. Further researches on calcium homeostasis and possible signal pathways are of great importance. In concluding, ECC act through complex, multicompound, multitarget and multipathway mechanisms in AD and might prove to be of great value in further clinical trials.

Animal experiments have contributed to our understanding of disease mechanisms, but the translation of preclinical experiments, which results in a prediction of the effectiveness of treatment strategies, to clinical trials is still challenging [81]. AD patients always have other medical problems such as aging, diabetes, hypertension and hyper lipidemia [82], and a gender difference is observed in AD prevalence [83]. The present study mainly included healthy male rats/mice, which may lead to selection bias to some extent. Registration of animal research prior to its execution in a generally accessible database similar to human (drug) research (www.clinicaltrials.com) may help to provide a more informed view before proceeding to clinical trials and may reduce publication bias [69,81].

### Conclusions

The present study showed ECC could improve cognitive function and exert potential neuroprotective effects in experimental AD, largely through mechanisms involving antioxidant stress and antiapoptosic effects, inhibiting Aβ deposition and tau protein hyperphosphorylation, and promoting synapse protection. Thus, ECC could be a candidate for further clinical trials of AD.

## Supplemenantry data

The Supplemenantry data can be found online at: http://dx.doi.org/10.14336/AD.2018.0815-1
