# Supplementary material for: Preclinical Evidence and Possible Mechanisms of Extracts or Compounds from Cistanches for Alzheimer’s Disease
Source: Aging Dis. 2019 Oct 1;10(5):1075–93. doi: 10.14336/AD.2018.0815-1 (PMC6764737; doi:10.14336/AD.2018.0815-1)
Supplement: Supplementary file 1 — The Supplemenantry data can be found online at: http://dx.doi.org/10.14336/AD.2018.0815-1 [file AD-10-5-1075-s.pdf]

SUPPLEMENTARY DATA

Preclinical Evidence and Possible Mechanisms of Extracts or Compounds from Cistanches for Alzheimer's Disease

Xiao-Li Zhou, Meng-Bei Xu, Ting-Yu Jin, Pei-Qing Rong, Guo-Qing Zheng*, Yan Lin*

Department of Neurology, the Second Affiliated Hospital and Yuying Children's Hospital of Wenzhou Medical University, Wenzhou, China

© 2018. Zhou XL et al. Published online at http://www.aginganddisease.org/EN/10.14336/AD.2018.0815-1

SUPPLEMENTARY DATA

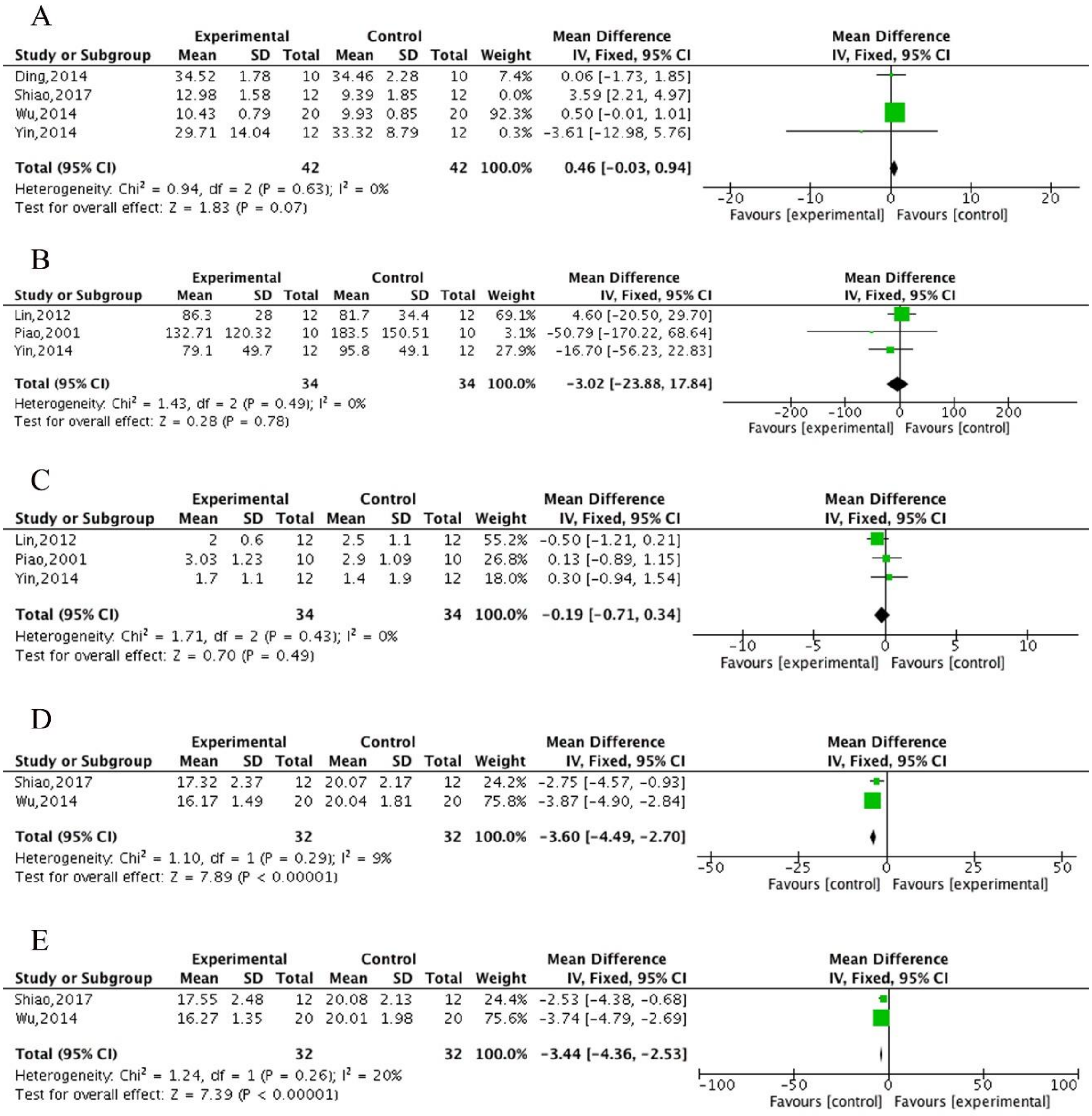

Supplemental Figure 1. The forest plot of neurobehavioral function indices changes. Effect of ECC for (**A**) increasing exact time in platform-quadrant in Morris water maze test, decreasing (**B**) react latency and (**C**) error times in retention test in Step-down test, increasing the (**D**) frequency and (**E**) time spending in Open field test compared with WCM controls.

© 2018. Zhou XL et al. Published online at http://www.aginganddisease.org/EN/10.14336/AD.2018.0815-1

SUPPLEMENTARY DATA

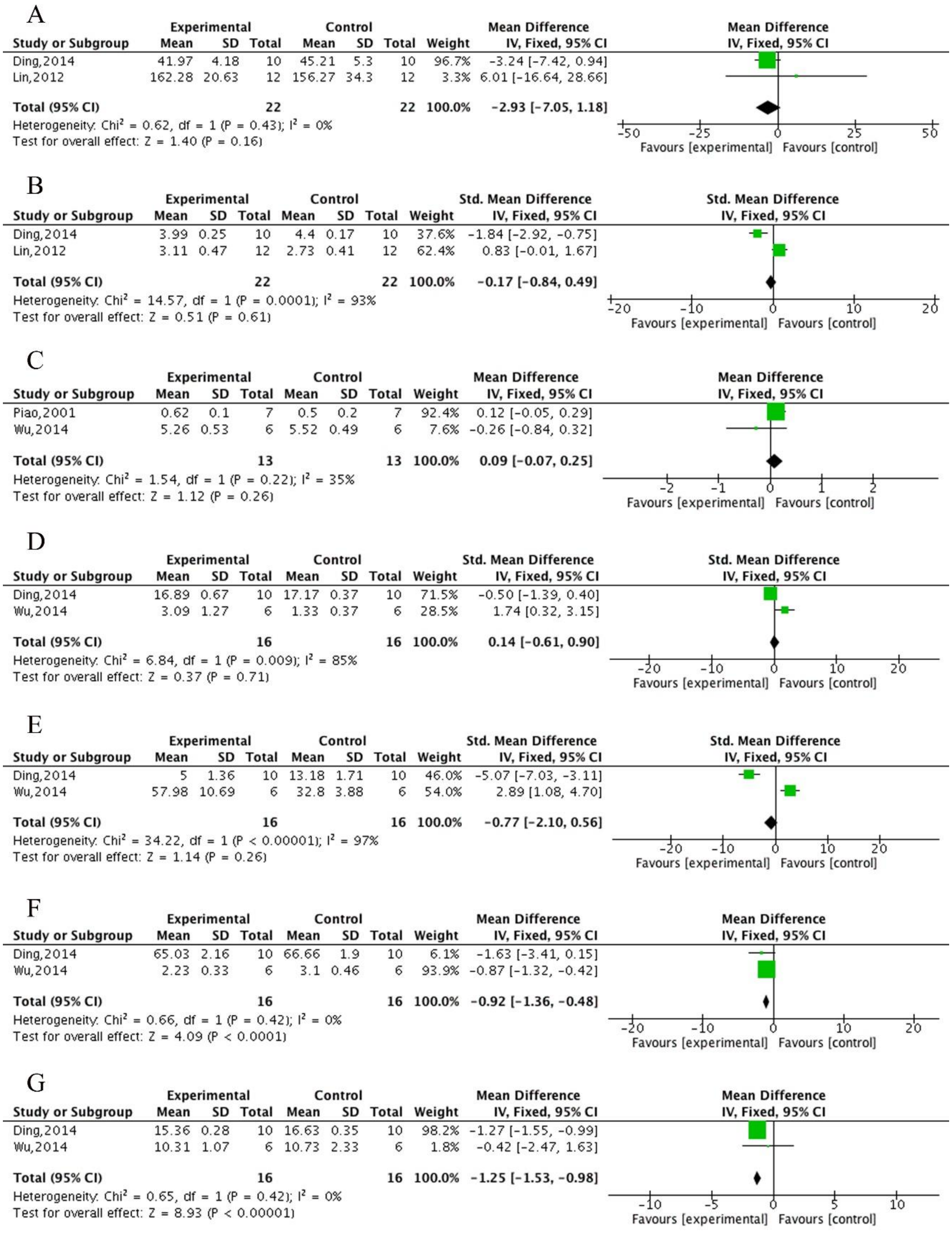

Supplemental Figure 2. The forest plot of neurobiochemical changes. Effect of ECC for increasing the activity of (**A**) SOD, (**B**) MDA and (**C**) AChE, increasing the level of DA and NE in hippocampus (**D** and **E**) and in cortex (**F** and **G**) compared with WCM controls.

© 2018. Zhou XL et al. Published online at http://www.aginganddisease.org/EN/10.14336/AD.2018.0815-1
